# Prenatal Diagnosis of Cystic Fibrosis by Celocentesis

**DOI:** 10.3390/genes15060662

**Published:** 2024-05-23

**Authors:** Antonino Giambona, Margherita Vinciguerra, Filippo Leto, Filippo Cassarà, Giuseppe Marchese, Valentina Cigna, Emanuela Orlandi, Maria Elena Mugavero, Gaspare Cucinella, Aurelio Maggio, Lisa Termini, George Makrydimas, Elena D’Alcamo, Francesco Picciotto

**Affiliations:** 1 Unit of Molecular Diagnosis of Rare Hematological Diseases, Azienda Ospedaliera Ospedali Riuniti Villa Sofia-Cervello, 90146 Palermo, Italy; m.vinciguerra@villasofia.it (M.V.); f.leto@villasofia.it (F.L.); cassarluca71@gmail.com (F.C.); giuseppemarchese1992@gmail.com (G.M.); elenadalcamo@gmail.com (E.D.); 2 Unit of Fetal Medicine and Prenatal Diagnosis, Azienda Ospedaliera Ospedali Riuniti Villa Sofia-Cervello, 90146 Palermo, Italy; valentinacigna@gmail.com (V.C.); orlandimanu78@gmail.com (E.O.); mugaveromariaelena@icloud.com (M.E.M.); gaspare.cucinella@unipa.it (G.C.); francescopicciotto64@gmail.com (F.P.); 3 Unit of Hematology for Rare Hematological Diseases, Azienda Ospedaliera Ospedali Riuniti Villa Sofia-Cervello, 90146 Palermo, Italy; aurelio.maggio@villasofia.it; 4 Ospedale dei Bambini G. Di Cristina, ARNAS Civico, 90134 Palermo, Italy; lisa.termini@arnascivico.it; 5 Department of Obstetrics & Gynecology, University of Ioannina, 45110 Ioannina, Greece; george.makrydimas@gmail.com

**Keywords:** celocentesis, prenatal diagnosis, celomatic fluid, fetal cells, recessive monogenic diseases, cystic fibrosis, β-Thalassemia

## Abstract

Celocentesis is a new sampling tool for prenatal diagnosis available from 7 weeks in case of couples at risk for genetic diseases. In this study, we reported the feasibility of earlier prenatal diagnosis by celocentesis in four cases of cystic fibrosis and one case of cystic fibrosis and β-thalassemia co-inherited in the same fetus. Celomic fluids were aspired from the celomic cavity between 8^+2^ and 9^+3^ weeks of gestation and fetal cells were picked up by micromanipulator. Maternal DNA contamination was tested and target regions of fetal DNA containing parental pathogenetic variants of *CFTR* and *HBB* genes were amplified and sequenced. Four of the five fetuses resulted as being affected by cystic fibrosis and, in all cases, the women decided to interrupt the pregnancy. In the other case, the fetus presented a healthy carrier of cystic fibrosis. The results were confirmed in three cases on placental tissue. In one case, no abortive tissue was obtained. In the last case, the woman refused the prenatal diagnosis to confirm the celocentesis data; the pregnancy is ongoing without complications. This procedure provides prenatal diagnosis of monogenic diseases at least four weeks earlier than traditional procedures, reducing the anxiety of patients and providing the option for medical termination of the affected fetus at 8–10 weeks of gestation, which is less traumatic and safer than surgical termination in the second trimester.

## 1. Introduction

To date, over 6000 different rare diseases have been identified, 75% of which are genetic, affecting approximately 30 million people in Europe and 300 million worldwide [1]. As they show a low prevalence in the world, they are defined as rare conditions. Cystic fibrosis and hemoglobinopathies (thalassemia and hemoglobin variants) are two of the most common serious monogenic diseases in the world [2,3]. Cystic fibrosis (MIM #219700) is the most common autosomal recessive fatal genetic disease, occurring in approximately 1 in 2500–3500 births among Caucasians and White Americans, 1 in 17,000 African Americans, and 1 in 31,000 Asian Americans [4]. According to the international registries, about 150,000 patients have a diagnosis of cystic fibrosis worldwide [5]. Cystic fibrosis results from pathogenetic variants in the transmembrane conductance regulator gene (*CFTR* MIM*602421) and appears to be clinically heterogeneous, spanning a wide phenotypic range in severity of symptoms [6,7,8].

The disease involves numerous organs and systems: the respiratory system, from the upper airways to the lung tissue; the pancreas for the production of digestive enzymes; the intestine causing malabsorption syndrome; and the reproductive system, especially the vase deferens in males.

In most cases, cystic fibrosis is diagnosed during childhood by using different tests that include newborn screening, sweat tests, genetic tests, lung function tests, nasal potential difference, and other tests [9,10]. β-thalassemia (MIM # 613985) is a common blood disorder worldwide. In 2008, the World Health Organization reported more than 40,000 new infants affected by β-thalassemia each year [11].

β-thalassemia occurs most frequently in people from Mediterranean countries, North Africa, the Middle East, India, Central Asia, and Southeast Asia [12,13,14,15]. Individuals with β-thalassemia major show pallor between 6 and 24 months of age due to severe anemia, poor weight gain, stunted growth, mild jaundice, and hepatosplenomegaly. Periodic red blood cell transfusions and iron chelation therapy permit normal growth and development and improve prognosis. Stunted growth, dilated cardiomyopathy, liver disease, and endocrinopathies are long-term complications associated with iron overload [16,17].

The increasing number of carrier screenings allows the identification of more couples with inheritable disease alleles. Management of pregnancies at risk of a genetic transmissible disorder has changed thanks to the prenatal diagnosis. Therefore, the demand for prenatal testing from individuals with at-risk pregnancies may increase. This procedure allows the couple to make a conscious choice about accepting a child affected by a genetically heritable pathology. The current laws and regulations in Italy, as in other European countries, allow prenatal diagnosis testing in cases of fetus with high risk of a severe genetic disease, such as cystic fibrosis or hemoglobinopathies [18]. Prenatal diagnosis is currently offered as part of public health programs if the parents are carriers of the same disease or if there is an affected member in the family. Currently, prenatal diagnosis can be performed either by non-invasive or invasive procedures [19,20,21]. Both types of procedures aim to detect previously characterized parental DNA pathogenetic variants in fetus when involving a couple at risk for a genetically transmissible disease. Non-invasive prenatal diagnosis involves the analysis of free fetal DNA (cffDNA) [22]. Free fetal DNA fragments are shorter than those of maternal cell-free DNA (cfDNA), and the size distribution is typically lower than 150 base pairs [23,24]. According to Ashoor et al., at 11 to 13 weeks of gestation, the fetal DNA fraction ranges from 7.8 to 13.0% and it is possible to obtain a useful result after 10 weeks of gestation [25].

In the case of monogenic disease, there are two mains limitation for the development of cffDNA-based prenatal tests: the low level of cffDNA present in maternal circulation and the exclusion of the paternal pathogenetic variant [26].

The identification of the paternal pathogenetic variant requires the exclusion of the maternal pathogenetic variant, and this requires an accurate evaluation of the hereditary defects of the fetus [27,28,29].

Invasive prenatal diagnosis for monogenic diseases has been well tested over the years. It can be performed by chorionic villus sampling (CVS) or amniocentesis. CVS is performed not earlier than 11 weeks to avoid miscarriage and development of limb defects [30,31]. Amniocentesis is performed not earlier than 16 weeks to avoid miscarriage and development of talipes [32]. The aspiration of celomatic fluid from the celomic cavity by celocentesis is an alternative technique for earlier diagnosis of monogenic diseases [33,34,35,36,37]. The celomic cavity inside the gestational sac reaches its maximum volume at 7–9 weeks and disappears at around 11–13 weeks [38]. This cavity acts as a possible reservoir for nutrients needed for the developing embryo [39]. The composition of celomic fluid is constituted by the ultrafiltrate of maternal serum with the addition of specific placental and secondary yolk sac proteins; it contains fetal cells coming from the yolk sac that can be sampled by celocentesis as early as 7–8 weeks [40,41]. Morphological and genetic studies have demonstrated that (i) the celomic fluid contains different cells of maternal and embryo–fetal origin; (ii) maternal contamination is high in most celomic fluid samples; (iii) erythroid precursor megaloblasts cells are the most common among the four types of identified embryo–fetal cells; (iv) maternal DNA contamination is solved, isolating embryo–fetal cells by picking up individually with a micromanipulator [42,43]. More than 700 prenatal diagnoses of hemoglobinopathies by celocentesis have been performed to date without any diagnostic errors and with a high degree of success [44,45]. In this study, we report for the first time the realization of a much earlier prenatal diagnosis by celocentesis at 8–9 weeks of gestation in four pregnancies at risk for cystic fibrosis and in one pregnancy at risk for both β-thalassemia and cystic fibrosis.

## 2. Materials and Methods

### 2.1. Patients

Four Sicilian couples and one Greek couple at risk for cystic fibrosis were asked to perform prenatal diagnosis by celocentesis. One couple presented risk for both β-thalassemia and cystic fibrosis. Preliminarily, the couple was counseled about the limited data on risks and diagnostic accuracy and advised to undergo conventional prenatal diagnosis (CVS or amniocentesis). The Institutional Review Board (IRB) approved the study and the couple provided written informed consent (Hospital Ethical Committee authorization on 26 January 2005, No 80). The advice from the Ethical Committee suggested that the diagnosis should be confirmed by CVS or amniocentesis. If the woman voluntarily decided to terminate the pregnancy in the event of an affected fetus, without having performed the control after the celocentesis, she was asked to examine the placental tissue after abortion. Informed consent was obtained with ethical approval from the Institutional Review Board, and genetic counseling was provided to the families.

### 2.2. Celocentesis: Sampling of Celomatic Fluid

Ampicillin (1 g) was administrated intramuscularly, about one hour prior to celocentesis. No local or general anesthesia was used. After disinfection of external genitalia, the gynecologists used a transvaginal 5 MHz ultrasound transducer that was covered with a sterile rubber. Fetal crown-rump length (CRL) and fetal heart rate were measured, and celomic space, amniotic membrane, and yolk sac were observed to check for any uterine anomalies. A 20 G needle was introduced transvaginally into the celomic cavity, and three consecutive samples of about 0.2, 0.2, and 0.6 mL were aspirated into three different syringes. The first and the second sample had a high number of red cells and maternal epithelial cells, while the third sample was less contaminated and considered to be the diagnostic sample. The fetal heart rate was measured again immediately after the procedure.

### 2.3. Fetal Cells Isolation

In a suitable environment, a biologist and a laboratory technician analyzed the third sample in order to minimize the risk of contamination. Samples were centrifuged at 3000 rpm for 7 min and supernatants were aspired. A total 100 μL of 0.9% NaCl was added to each sample and placed in a petri dish. Embryo–fetal erythroid precursor cells were identified by inverted optical phase contrast microscopy using a Leika AM 6000 microscope (Leica Microsystems, Wetzlar, Germany) at 40× magnification. A micromanipulator (Eppendorf–Leika AM 6000, Hamburg, Germany) with a 45 μm glass micropipette (Cook Medical, Limerick, Ireland) was used for aspiration of about 20 fetal cells placed in a drop of 0.9% NaCl in the same petri dish.

#### 2.3.1. DNA Extraction

Fetal cells, aspired from petri dish, were put into a tube with 4 μL of 0.05 M NaOH, 0.5 μL of 1% triton, and 1 μL of 12.5 μm Protease K and incubated at 45 °C for 10 min and 94 °C for 10 min. A total 1 μL of 1 M TrisHCl pH 8.0 was added to each sample. Maternal DNA was extracted by salting-out procedure [46].

#### 2.3.2. DNA Amplification

Targets sites of *CFTR* gene (GenBank LRG_663) and *HBB* gene (GenBank LRG_1232) containing pathogenetic variants were amplified using specific primers for PCR (Table S1, Supplementary Materials). Maternal contamination was checked by quantitative fluorescent PCR (QF-PCR) procedure [47]. After the first step of multiplex PCR, internal primers were used to perform specific nested PCR because of the low amount of fetal DNA.

#### 2.3.3. First Step of Amplification

Specific primers for detection of parental pathogenetic variants in *CFTR* and *HBB* genes were used in 50 µL reaction volume with a mix of primers to amplify many short tandem repeats (STR) located on chromosomes 13, 18, and 21 X and Y for checking maternal DNA contamination. A total 25 µL of 2X Platinum Multiplex PCR Master Mix (Life Technologies, Carlsbad, CA, USA), 10 pm/µL of each forward and reverse primer to amplify *CFTR* gene (the same amount of primers for *HBB* amplification in the case of the couple at risk both for *CFTR* and *HBB* defects), 3–10 pmol of each forward and reverse primer for STR, and 11.5 μL of distilled water were added into the tubes containing lysed cells. PCR was performed in a Gene Ampl 9700, Applied Biosystems, Foster City, CA, USA (1 cycle of denaturation at 95 °C for 2′; 25 cycles at 95 °C 30″, 60 °C 90″, and 72 °C 60″; and a final cycle at 72 °C for 30′).

#### 2.3.4. Nested PCR

*CFTR*, *HBB* genes, and STR markers were amplified using inner primers in different nested PCR performed in a 30 µL reaction volume (Table S1, Supplementary Materials).

### 2.4. Nested PCR for CFTR and HBB Genes

Fragments containing pathogenetic variants of *CFTR* and *HBB* genes were amplified in different tubes. 3 µL of the first multiplex PCR product, 15 µL of 2X Platinum Multiplex PCR Master Mix, 10 pm/µL of specific *CFTR* or *HBB* inner primers and distilled water were added into new tubes (1 cycle of denaturation at 95 °C for 2′; 25 cycles at 95 °C 30″, 60 °C 90″, and 72 °C 60″; and a final cycle at 72 °C for 7′). Amplified products were sequenced with specific oligonucleotides (Table S1, Supplementary Materials) and the BigDye Terminator 3.1 Cycle Sequencing kit (Applied Biosystems, Foster City, CA, USA) and run on ABI PRISM 3130 xl genetic DNA analyzer (Applied Biosystems).

### 2.5. Nested Quantitative Fluorescent PCR (QF-PCR) for Maternal Contamination Evaluation

**Figure 1:**
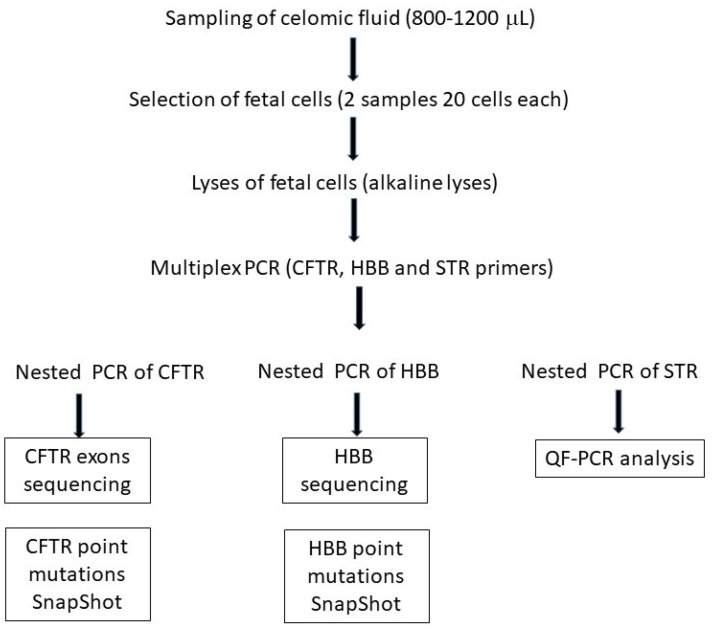
Workflow for analysis of celomatic fluid. Forty fetal cells are isolated by a micromanipulator and equally distributed in two 0.2 mL Eppendorf tubes. Fetal cells are lysed by alkaline procedure. Initially, a multiplex Polymerase chain reaction (PCR) using CFTR, HBB and STR primers is performed. Subsequently, specific nested PCR for CFTR and HBB target regions and STR are separately performed. Sequencing and SnapShot analysis are performed to evaluate the presence of parental mutations. Fragment analysis is used to evaluate the presence of maternal contamination.

A total 3 µL of first multiplex PCR product, 15 µL of 2X Platinum Multiplex PCR Master Mix, and 3–10 pmol of labeled primers and distilled water were used for STR amplification (1 cycle of denaturation at 95 °C for 2′; 25 cycles at 95 °C 30″, 60 °C 90″, and 72 °C 60″; and a final cycle at 72 °C for 30′). Primers labeled with different fluorochromes allow analysis of amplicons in the same multiplex QF-PCR reactions. A total 2.5 μL of PCR products were mixed with 15 μL of formamide and 0.4 μL of GeneScan-500 Liz Size Standard. Capillary electrophoresis for fluorescent amplicons was performed with ABI PRISM 3130 xl genetic analyzer automated DNA sequencer and GeneScan 4.0 software analyzer (Applied Biosystems, Foster City, CA, USA). QF-PCR data on celomic fluid were compared with parental DNA results. In Figure 1, the laboratory workflow for prenatal diagnosis using celomic fluid cells is reported.

## 3. Results

### 3.1. Sampling of Celomatic Fluid

Celocentesis was successfully performed in all of the women, and an adequate sample (800–1200 µL) of celomic fluid was obtained in all cases.

### 3.2. Fetal Cell Isolation

An adequate number of cells was observed in all the celomic fluids. Forty fetal erythroblast cells were isolated and equally distributed (*n* = 20) in two different 0.2 mL Eppendorf tubes (Figures S1–S5, Supplementary Materials).

### 3.3. Maternal Contamination Evaluation

No maternal contamination was found by QF-PCR analysis in all five celomic samples of fetal cells selected with the micromanipulator. The ratio between fluorescent peak areas of maternal and paternal origin was about 1:1 (range 1.0–1.3). No triallelic peaks or skewed diallelic data were present (Figures S1–S5, Supplementary Materials).

### 3.4. CFTR Analysis

#### 3.4.1. Case 1

The celomic fluid was sampled in Greece and sent to our laboratory. The Greek, non-consanguineous couple at risk for cystic fibrosis showed the same pathogenetic variant in the exon 11 of *CFTR* gene NM_000492.4:c.1521_1523delCTT (Phe508del).

This pathogenetic variant results in a lack of the amino acid phenylalanine at position 508 in the *CFTR* protein and is reported as a disease-causing variant (ClinVar, HGMD). Celocentesis was performed at 9^+0^ weeks of gestation, and 1250 µL of celomic fluid was aspired, in absence of evident ultrasound signs.

As is visible in Figure S1 (Supplementary Materials), forty cells were recovered and distributed into two tubes containing twenty cells each (A). Paternal, fetal, and maternal QF-PCR electropherograms showed the absence of maternal contamination confirmed by the different STR profiles between the mother and the celomic fluid sample. The fetus was a male (B). The specific *CFTR* pathogenetic variant in the celomic fetal DNA was identified in the homozygous state and the fetus was diagnosticated as being affected by cystic fibrosis (C). The SnapShot molecular analysis of *CFTR*:c.1521_1523delCTT confirmed the results obtained by sequencing (D).

Analysis of placental tissue after voluntary interruption of pregnancy (VIP), performed at the 10th week after genetic counseling, was not carried out to confirm the result.

#### 3.4.2. Case 2

A non-consanguineous Sicilian couple was at risk for β-thalassemia and cystic fibrosis; therefore, the risk of disease transmission was increased. The father had *HBB*:c.92-21G>A and *CFTR*:c.350G>A pathogenetic variants, while the mother showed *HBB*:c.-137C>G and *CFTR*:c.1521_1523delCTT pathogenetic variants.

Celocentesis was performed at 8^+4^ weeks of gestation and 1100 µL of celomic fluid were aspired, in absence of evident ultrasound signs. As is visible in Figure S2 (Supplementary Materials), forty cells were recovered and distributed into two tubes containing twenty cells each (A). Paternal, fetal, and maternal QF-PCR electropherograms showed the absence of maternal contamination confirmed by the different STR profiles between the mother and the celomic fluid sample. The fetus was a female (B). The fetus showed only the paternal *HBB*:c.92-21G>A pathogenetic variant (C and D) and both parental *CFTR* pathogenetic variants, c.350G>A and c.1521_1523delCTT, identified by sequencing analysis (E and F). SnapShot tests confirmed the data (G and H). Fetus resulted as a health carrier for β-thalassemia but affected by cystic fibrosis.

After genetic counseling, the patient refused to undergo the control by CVS or amniocentesis and required voluntary interruption of pregnancy at 9^+5^ weeks. The results were confirmed on placental tissue after VIP.

#### 3.4.3. Case 3

A Sicilian non-consanguineous couple was at risk for cystic fibrosis. They had previously had an affected daughter. The parental *CFTR* pathogenetic variants identified were *CFTR*:c.3230T>C in the father and *CFTR*:c.1521_1523delCTT in the mother. Celocentesis was performed at 8^+2^ weeks of gestation and 1250 µL of celomic fluid was aspired, in absence of evident ultrasound signs. As it is visible in Figure S3 (Supplementary Materials), forty cells were recovered and distributed into two tubes containing twenty cells each (A). Paternal, fetal and maternal QF-PCR electropherograms showed the absence of maternal contamination confirmed by the different STR profile between the mother and the celomic fluid sample. The fetus was a female (B). Analysis of fetal DNA of exons 11 and 20 of the *CFTR* gene, by sequencing, showed the presence of both parental pathogenetic variants (C and D). SnapShot tests confirmed the data (E and F). The fetus was found to be affected by cystic fibrosis.

After genetic counseling, the patient refused to undergo the control by CVS or amniocentesis and required voluntary interruption of pregnancy at 9^+4^ weeks. The results were confirmed on placental tissue after VIP.

#### 3.4.4. Case 4

A Sicilian non-consanguineous couple was at risk for cystic fibrosis. The father showed the *CFTR*:c.3909C>G pathogenetic variant and the mother the *CFTR*:c.1657C>T pathogenetic variant. Previously, the woman had undergone a prenatal diagnosis using CVS with the result of a healthy fetus. Celocentesis was performed at 8^+5^ weeks of gestation and 1100 µL of celomic fluid was aspired, in absence of evident ultrasound signs. As is visible in Figure S4 (Supplementary Materials), forty cells were recovered and distributed into two tubes containing twenty cells each (A). Paternal, fetal, and maternal QF-PCR electropherograms showed the absence of maternal contamination confirmed by the different STR profile between the mother and the celomic fluid sample. The fetus was a female (B). Analysis of fetal DNA of exons 12 and 24 of the *CFTR* gene, by sequencing, showed the presence of both parental pathogenetic variants (C and D). These data were confirmed by SnapShot tests (E and F). The fetus was found to be affected by cystic fibrosis. After genetic counseling, the patient refused to undergo the control by CVS or amniocentesis and required voluntary interruption of pregnancy at 9^+6^ weeks. The results were confirmed on placental tissue after VIP.

#### 3.4.5. Case 5 CFTR Analysis

A Sicilian couple carrier of cystic fibrosis had a previous affected daughter. Parental pathogenetic variants identified were *CFTR*:c.1624G>T (G542X) in the father and *CFTR*:c.1521_1523delCTT (F508del) in the mother. Celocentesis was performed in January 2024 at 9^+3^ weeks of gestation and 1150 µL of celomic fluid was aspired in absence of evident ultrasound signs. DNA analysis showed no maternal DNA contamination. As is visible in Figure S5 (Supplementary Materials), forty cells were recovered and distributed into two tubes containing twenty cells each (A). Paternal, fetal, and maternal QF-PCR electropherograms showed the absence of maternal contamination confirmed by the different STR profiles between the mother and the celomic fluid sample. The fetus was a female (B). Only the maternal pathogenetic variant was identified; thus, the fetus resulted as a healthy carrier of cystic fibrosis (C and D). These data were confirmed by SnapShot tests (E and F). After genetic counseling, the patient refused to undergo control by CVS or amniocentesis.

To date, the pregnancy is ongoing without any complication.

The results of the analyses of the five cases are summarized in Table 1

## 4. Discussion

### 4.1. Potentialities of Procedure

Invasive prenatal diagnosis of monogenic disease is performed by chorionic villus or amniotic liquid sampling after the 11th or 16th weeks of gestation. Early prenatal diagnosis is important to reduce anxiety and allow the possibility of VIP in case of an affected fetus, in consideration of the serious implications of the disease and the probability (25%) when both parents are carriers. Celocentesis can be successfully used for the determination of fetal sex, sickle cell anemia, and thalassemia from celomic cells [31,42]. It was reported that human celomic fluid contains embryonic erythroid precursor cells [40,41], and the feasibility of prenatal diagnosis of hemoglobinopathies was demonstrated in more than 500 cases [42]. There is a growing interest in earlier prenatal testing for diagnosis of monogenic diseases in the Italian population, and celocentesis allows it at least four weeks earlier than traditional procedures.

This study reported the suitability of celomic fluid for very early prenatal diagnosis of monogenic diseases, isolating single fetal cells by a micromanipulator even in the presence of a high degree of maternal contamination. We have reported the application of celocentesis in five couples at risk for cystic fibrosis, of which one couple is at risk for both cystic fibrosis and beta thalassemia, and we have demonstrated for the first time that it is possible to perform very early prenatal diagnosis of cystic fibrosis using fetal cells present in the celomic fluid. Further, we have demonstrated that it is possible to perform molecular analysis in cases of couples at risk for two transmissible monogenic diseases and to control maternal DNA contamination. In addition, the celomic fluid sampled in Greece arrived at our laboratory after 48 h, confirming the possibility to send celomic fluid while maintaining the cells’ integrity for molecular analysis.

The sensitivity and specificity of the procedure were very high, as demonstrated by analysis of placental tissue after VIP in three of the five reported cases, which was performed after genetic counseling, confirming the results obtained on celomic fluid.

In one case, it was not possible to obtain the aborted tissue, and in the other case, the patient refused control with other invasive procedures and the pregnancy is still ongoing without complications. The other point of interest of celocentesis concerns the possibility of obtaining the fetal karyotype in a very early period [43]. Today, the use of analysis of cell-free DNA in maternal blood is increasingly frequent; this technique provides screening for trisomies 21, 18, and 13 without any risk of fetal loss. However, this procedure is carried out after 10 weeks of gestation in order to minimize failure rate due to low fetal fraction. Cell-free DNA testing is another procedure used only in the case of different parental pathogenetic variants; otherwise, the only available options for prenatal diagnosis remain, to date, CVS or amniocentesis. Celocentesis may be an earlier valid alternative to CVS and amniocentesis, particularly in areas where specific monogenic diseases are present at a high frequency, such as hemoglobinopathy in the Mediterranean area. Furthermore, pregnancy termination is less risky in the first trimester of gestation than in later weeks, in case the patient prefers to undergo VIP. Another very important aspect is genetic counseling. This aspect represents a critical issue, especially in the case of a couple at risk for two different genetic diseases, as it is more complex to communicate with the couple. In this case, careful genetic counseling is mandatory, and a broader approach is strongly needed that takes into account the couple’s discomfort in learning that the fetus is healthy for one disease and affected by the other, which can give rise to hope for a more favorable outcome in the future.

In most countries, the deadline for voluntary interruption of pregnancy is 12 weeks. The possibility of carrying out prenatal diagnosis at such an early stage of pregnancy is important, above all, from a psychological point of view for the couple undertaking this path. In fact, the practice of therapeutic abortion carried out at 9/10 weeks of gestation is comparable to that of voluntary interruption of pregnancy carried out within 90 days in most countries in the world and, in some cases, with much less invasive procedures such as pharmacological treatment, which can be carried out in Italy up to 63 days into gestation. Furthermore, celocentesis can be used for all serious monogenic pathologies and allows finding the presence of DNA pathogenetic variants with an accuracy (according to our current data) of 100%.

### 4.2. Limitations of Procedure

The presence of maternal cell contamination was the main problem of this early procedure [35], but it has been overcome by the optimization of a laboratory workflow, even in low-cellular samples [47]. The absence of cells of fetal origin in celomic fluid remain the principal limitation of celocentesis, but it rarely happens (<1%). The training of experienced gynecologists capable of carrying out the sampling procedure is a fundamental starting point and is quite easily achieved.

The recognition of fetal cells present in the celomatic fluid and their selection by experienced laboratory personnel is perhaps the main critical point that is limiting the spread of this procedure. Further studies are underway to automate this delicate phase, taking advantage of the fact that most of the fetal cells in the celomatic fluid are embryo–fetal erythroid precursors that express the CD71 antigen on the cell membrane.

This study reported a reduced number of cases but further applications of this procedure to prenatal diagnosis of cystic fibrosis are underway. Furthermore, we must consider that more than 700 prenatal diagnoses of hemoglobinopathies by celocentesis have already been performed to date without any diagnostic errors and with no complications in the course of pregnancy [44,45].

## 5. Conclusions

Celocentesis can be used for early prenatal diagnosis of cystic fibrosis and beta thalassemia, and for other monogenic genetic disease too [48,49,50]. Thanks to its earliness, celocentesis is a valid alternative to CVS or amniocentesis because it provides an earlier diagnosis and less-traumatic termination of pregnancy if the patient prefers to undergo voluntary interruption of pregnancy in case of an affected fetus.

## Figures and Tables

**Table 1:** Results of the analysis of the 5 cases.

	Sampling Week	Sampling Amount (μL)	Paternal Mutation	Maternal Mutation	Fetal Genotype	Fetal Condition	Post Celocentesis Control	Voluntary Interruption of Pregnancy
CASE 1	9^+0^	1250	CFTR:c.1521_1523delCTT	CFTR:c.1521_1523delCTT	CFTR:c.[1521_1523delCTT];[1521_1523delCTT]	Affected of cystic fibrosis	NO	YES at 10^+0^
CASE 2	8^+4^	1100	CFTR:c.350G>A	CFTR:c.1521_1523delCTT	CFTR:c.[350G>A];[1521_1523delCTT]	Affected of cystic fibrosis	YES on abortive tissue	YES at 9^+5^
			HBB:C.92-21G>A	HBB:c.-137C>G	HBB:c.[92-21G>A];[=]	healthy carrier of β-thalassemia		
CASE 3	8^+2^	1250	CFTR:c.3230T>C	CFTR:c.1521_1523delCTT	CFTR:c.[3230T>C];[1521_1523delCTT]	Affected of cystic fibrosis	YES on abortive tissue	YES at 9^+4^
CASE 4	8^+5^	1100	CFTR:c.3909C>G	CFTR:c.1657C>T	CFTR:c.[3909C>G];[1657C>T]	Affected of cystic fibrosis	YES on abortive tissue	YES at 9^+6^
CASE 5	9^+3^	1150	CFTR:c.1624G>T	CFTR:c.1521_1523delCTT	CFTR:c.[1521_1523delCTT];[=]	healthy carrier of cystic fibrosis	NO	Pregnancy on going

## Data Availability

The original contributions presented in the study are included in the article and Supplementary Materials, further inquiries can be directed to the corresponding author.

## Supplementary Materials

The following supporting information can be downloaded at: https://www.mdpi.com/article/10.3390/genes15060662/s1, Figure S1. Case 1; Figure S2. Case 2; Figure S3. Case 3; Figure S4. Case 4; Figure S5. Case 5; Table S1. Sequence of primers.
