# Supplementary material for: Prenatal Diagnosis of Cystic Fibrosis by Celocentesis
Source: Genes (Basel). 2024 May 23;15(6):662. doi: 10.3390/genes15060662 (PMC11203072; doi:10.3390/genes15060662)
Supplement: Supplementary file 1 [file genes-15-00662-s001.zip › Figure S4.pdf]

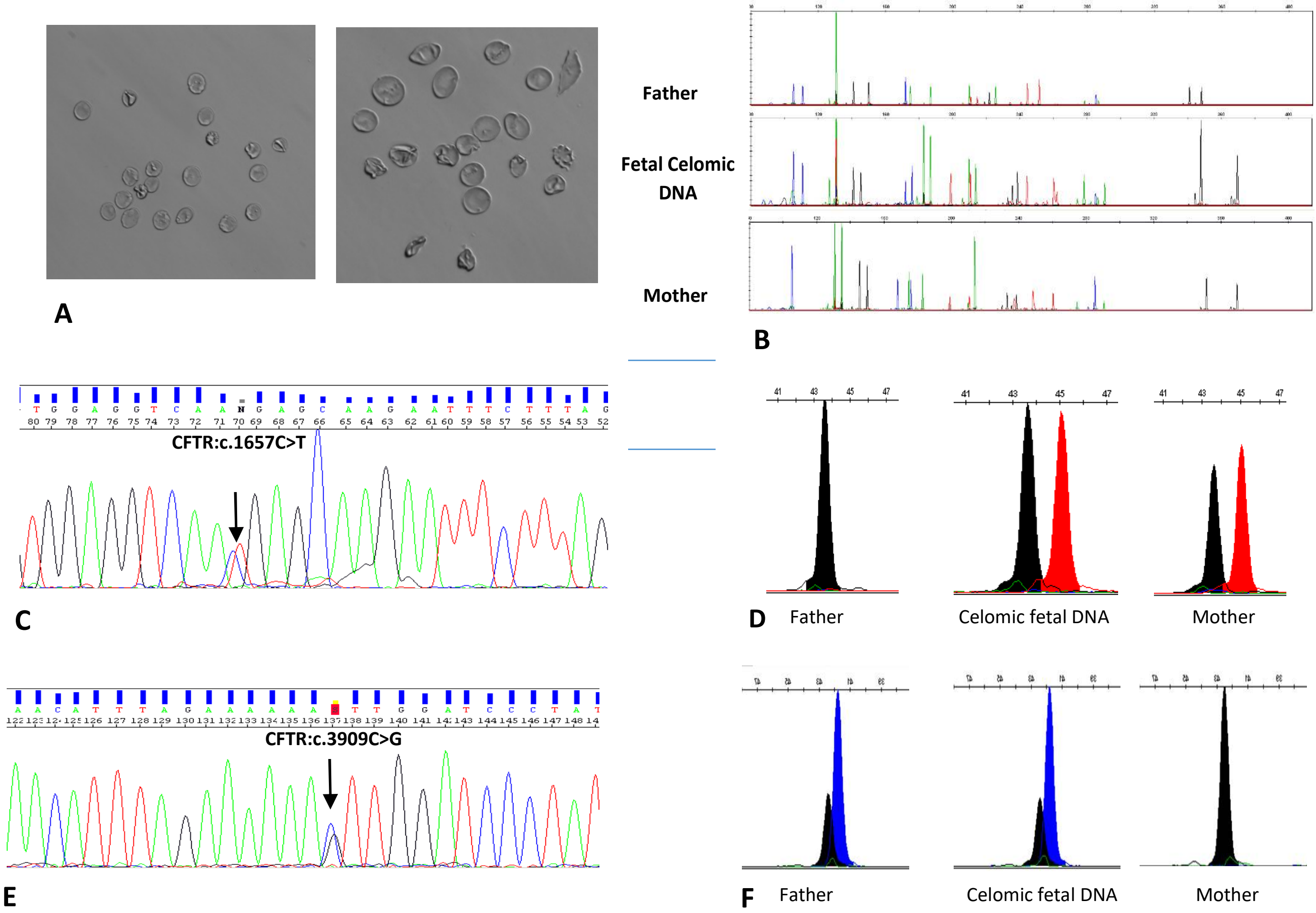

Figure S4. Case 4.
