# Supplementary material for: Prenatal Diagnosis of Cystic Fibrosis by Celocentesis
Source: Genes (Basel). 2024 May 23;15(6):662. doi: 10.3390/genes15060662 (PMC11203072; doi:10.3390/genes15060662)
Supplement: Supplementary file 1 [file genes-15-00662-s001.zip › Table S1.pdf]

CFTR GENE	SEQUENCE
exon 4 Forward	TCCCAAGTCTTATTTCAAAGTACC
exon 4 Reverse	GAGCATTAATTATTCCTGCCATTTA
exon 4 Forward nested	AGTGCTAAGAGTTTCACATATGG
exon 4 Reverse Nested	ATTTCAGCATTTATCCCTTACTTG
exon 4 Forward sequencing	AACTCATTTTAAGTCTCCTCTAAAG
exon 4 Reverse sequencing	GCTCACTACCTAATTTATGACATT
SnapShot c.350G>A	(22T)-GACCCGGATAACAAGGAGGAAC
exon 11 Forward	TGAATCATGTGCCCCTTCTCTG
exon 11 Reverse	CATTTATTGTGATCAAATGAACCC
exon 11 Forward nested	GTGCATAGCAGAGTACCTGAAACCC
exon 11 Reverse Nested	TTATTTCATGTGTTTGCAAGCTTC
exon 11 Forward sequencing	TGAGTTAATAGAATCTTTACAAATAAG
exon 11 Reverse sequencing	TTCACAGTAGCTTACCCATAGAGG
SnapShot c.1521_1525delCTT	(22T)-GCCTGGCACCATTAAAGAAAATATCAT
exon 12 Forward	CAGCAATGTTGTTTTTGACCAAC
exon 12 Reverse	ACCATAATCTCTACCAAATCTGG
exon 12 Forward nested	GAAATAATGGAGATGCAATGTTCA
exon 12 Reverse Nested	CACTAGCCATAAAACCCCAGGA
exon 12 Forward sequencing	AACTGTGGTTAAAGCAATAGTGTG
exon 12 Reverse sequencing	GCAATAGAGAAATGTCTGTAATT
SnapShot c.1657C>T	(22T-GAATCACACTGAGTGGAGGTCAA
SnapShot c.1624G>T	(22T)-GCAGAGAAAGACAATATAGTTCTT
exon 20 Forward	TTCAAAGAATGGCACCAGTGTG
exon 20 Reverse	CACTTGTTCAATAATAAACCAAC
exon 20 Forward nested	CCAATGACATTTGTGATATGATTATT
exon 20 Reverse Nested	CCTATAGAATGCAGCATTTTATTC
exon 20 Forward sequencing	ATTTAGTCTTTTTCAGGTACAAGA
exon 20 Reverse sequencing	CTTAAATGCTTAGCTAAAGTTAATG
SnapShot c.3230T>C	(22T)-GACGGCAGCCTTACTTTGAAACTC
exon 24 Forward	GAATGATACAAAGCAGACATGATA
exon 24 Reverse	CTCCATTTTGAAGTTGTGCACAG
exon 24 Forward nested	CTTGATGGTAAGTACATGGGTGT
exon 24 Reverse Nested	CACACAAAGTGTGTAGAATGATC
exon 24 Forward sequencing	CTACTTGAAATATTTTACAATAC
exon 24 Reverse sequencing	TCAACCATTTGTGTTGGTATGAG
SnapShot c.3908C>G	(22T)-TTTTCTGGAACATTTAGAAAAAA

**HBB GENE**	**SEQUENCE**
Forward	GTAAATACACTTGCAAAGGAGGATG
Reverse	CAATGTTAAGGCATTAAGTATAATAG
Forward nested	AGAGATATATCTTAGAGGGAGGG
Reverse Nested	GAAAACAATTGTTATGAACAGCA
Forward sequencing	TTTGAAGTCCAACTCCTAAGC
Reverse sequencing	CATTCGTCTGTTTCCCATTCTAAA
SnapShot c.-137C>G	(53T)-AGACCTCACCCTGTGGAGCCACAC
SnapShot c.93-21G>A	(10T)-AGGCACTGACTCTCTCTGCCTATT
