# Supplementary figures and images for: Prenatal Diagnosis of Cystic Fibrosis by Celocentesis
Source: Genes (Basel). 2024 May 23;15(6):662. doi: 10.3390/genes15060662 (PMC11203072; doi:10.3390/genes15060662)

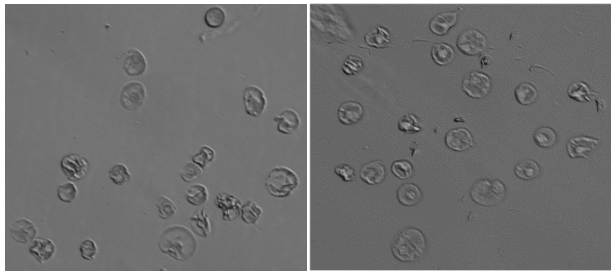

**A**

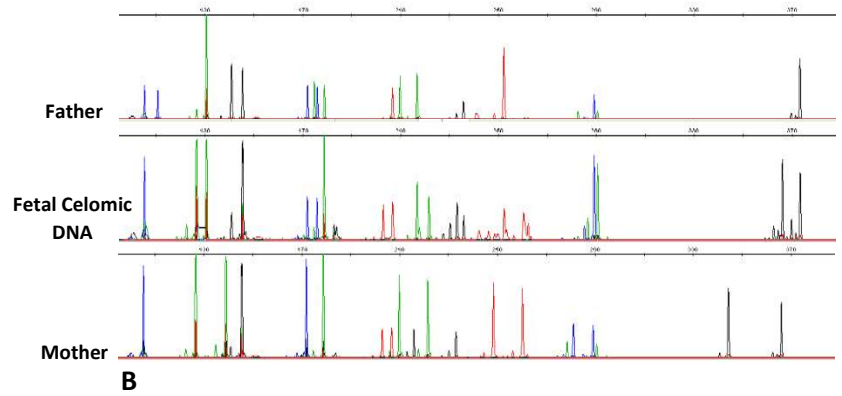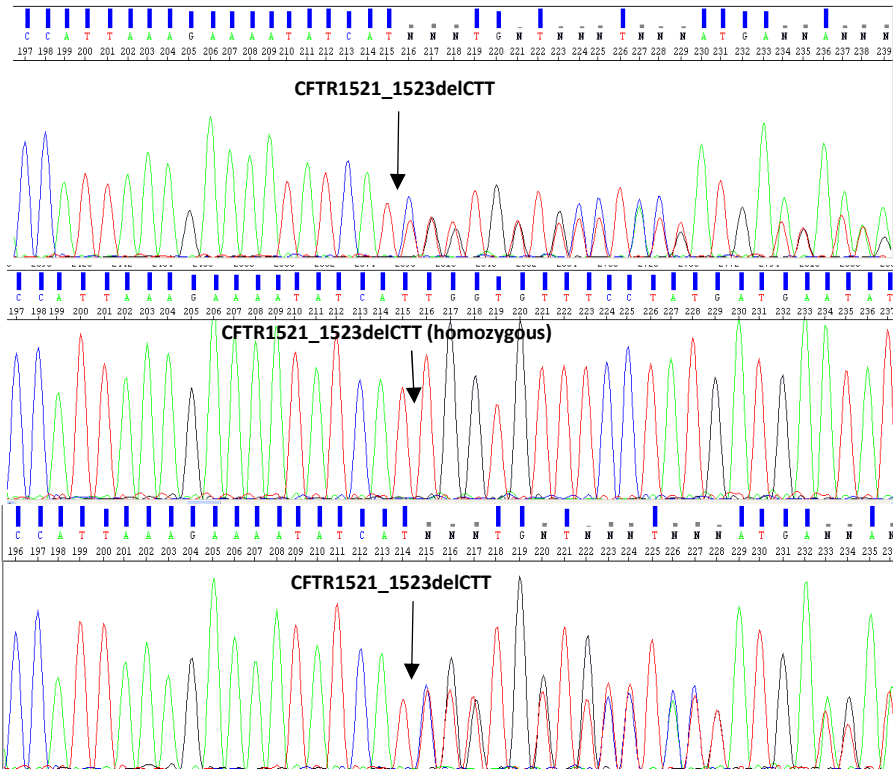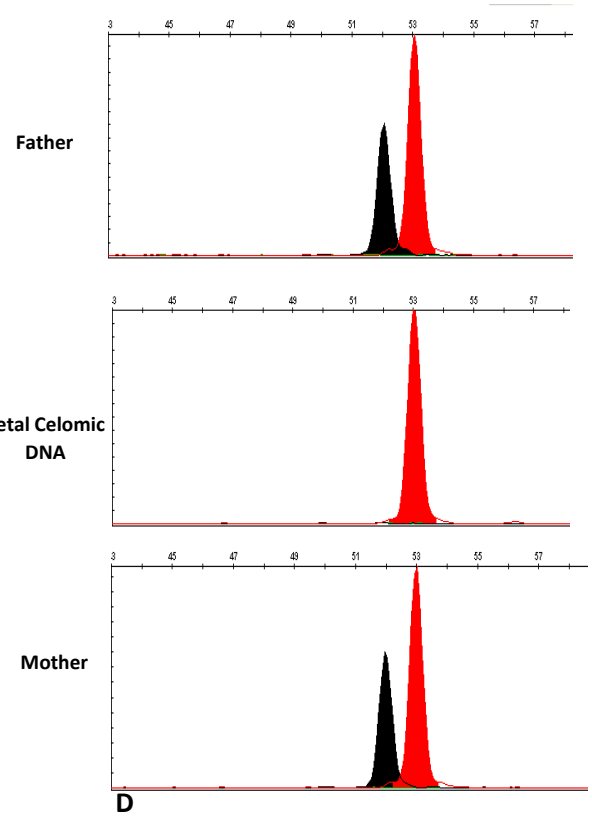

**Figure S1. Case 1.**

Supplement: Supplementary file 1 [file genes-15-00662-s001.zip › Figure S1.pdf]

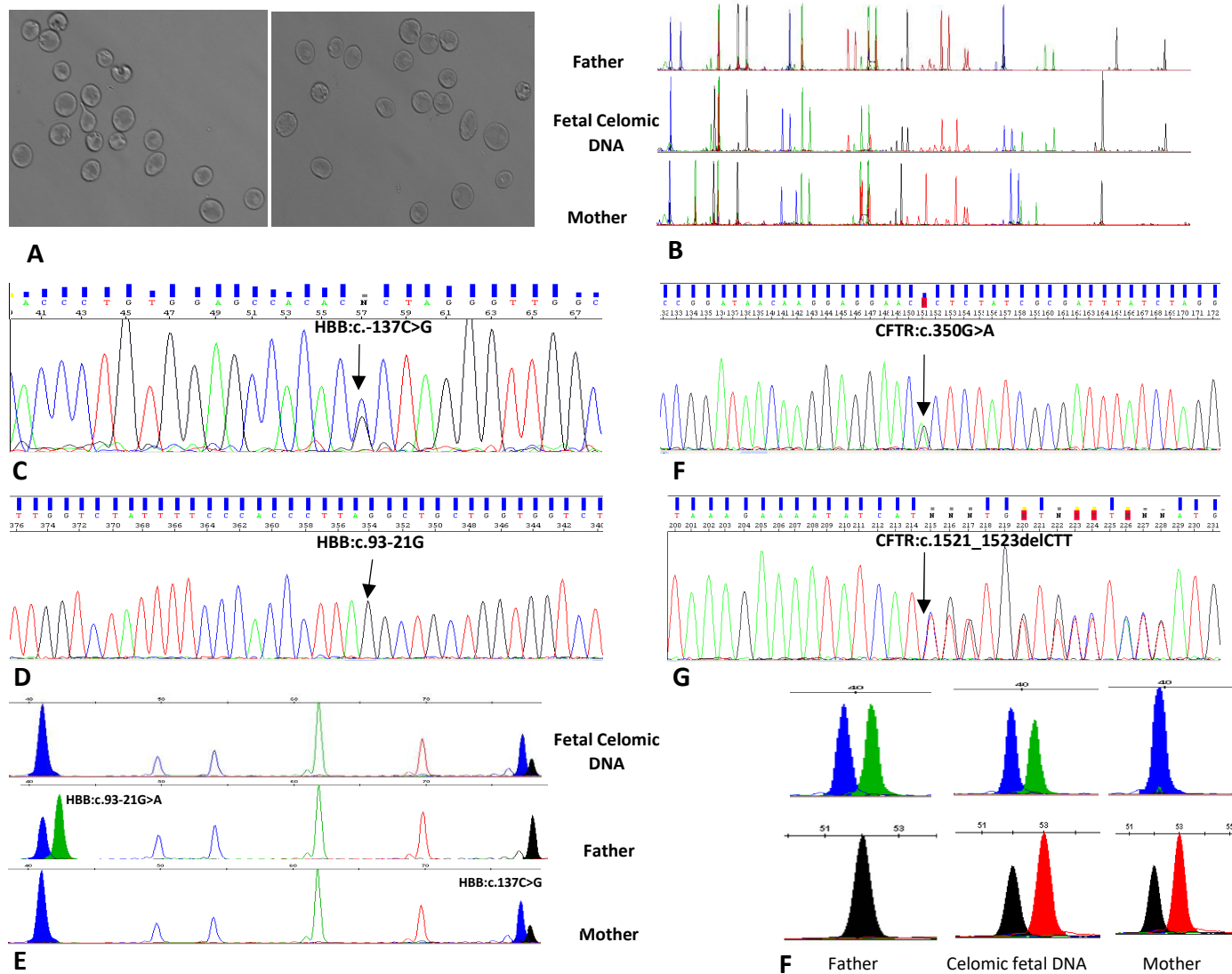

**Figure S2. Case 2.**

Supplement: Supplementary file 1 [file genes-15-00662-s001.zip › Figure S2.pdf]

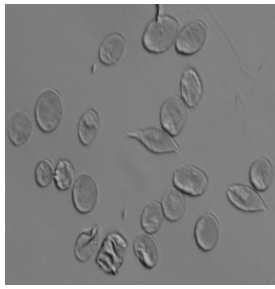

**A**

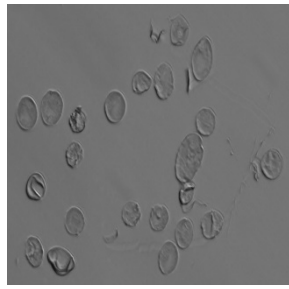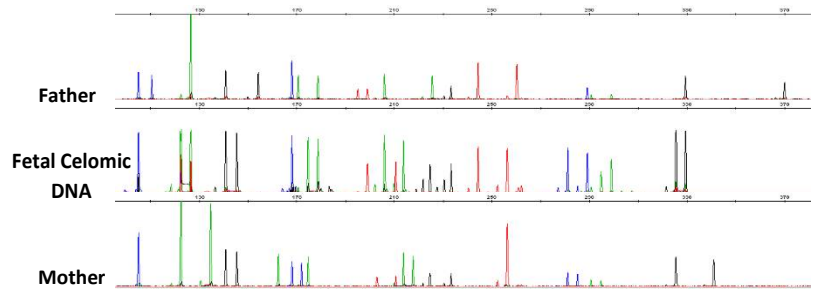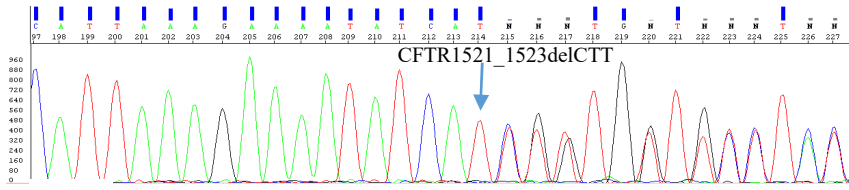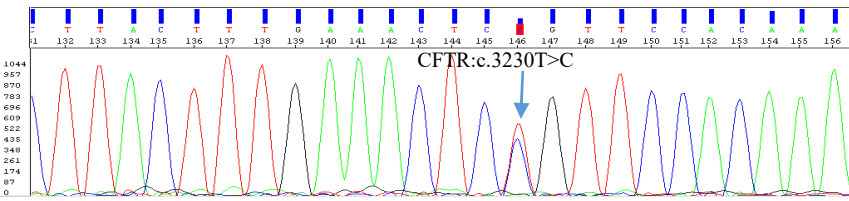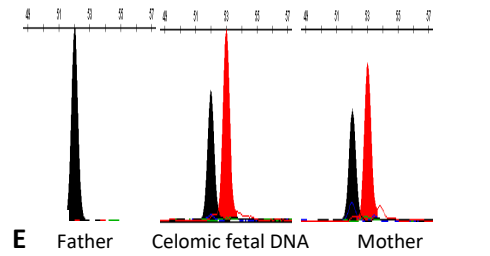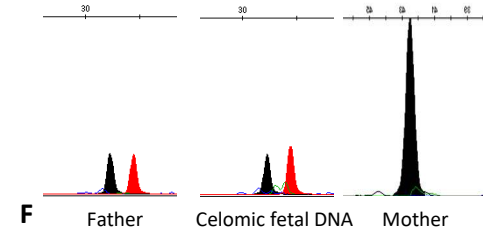

**Figure S3. Case 3.**

Supplement: Supplementary file 1 [file genes-15-00662-s001.zip › Figure S3.pdf]

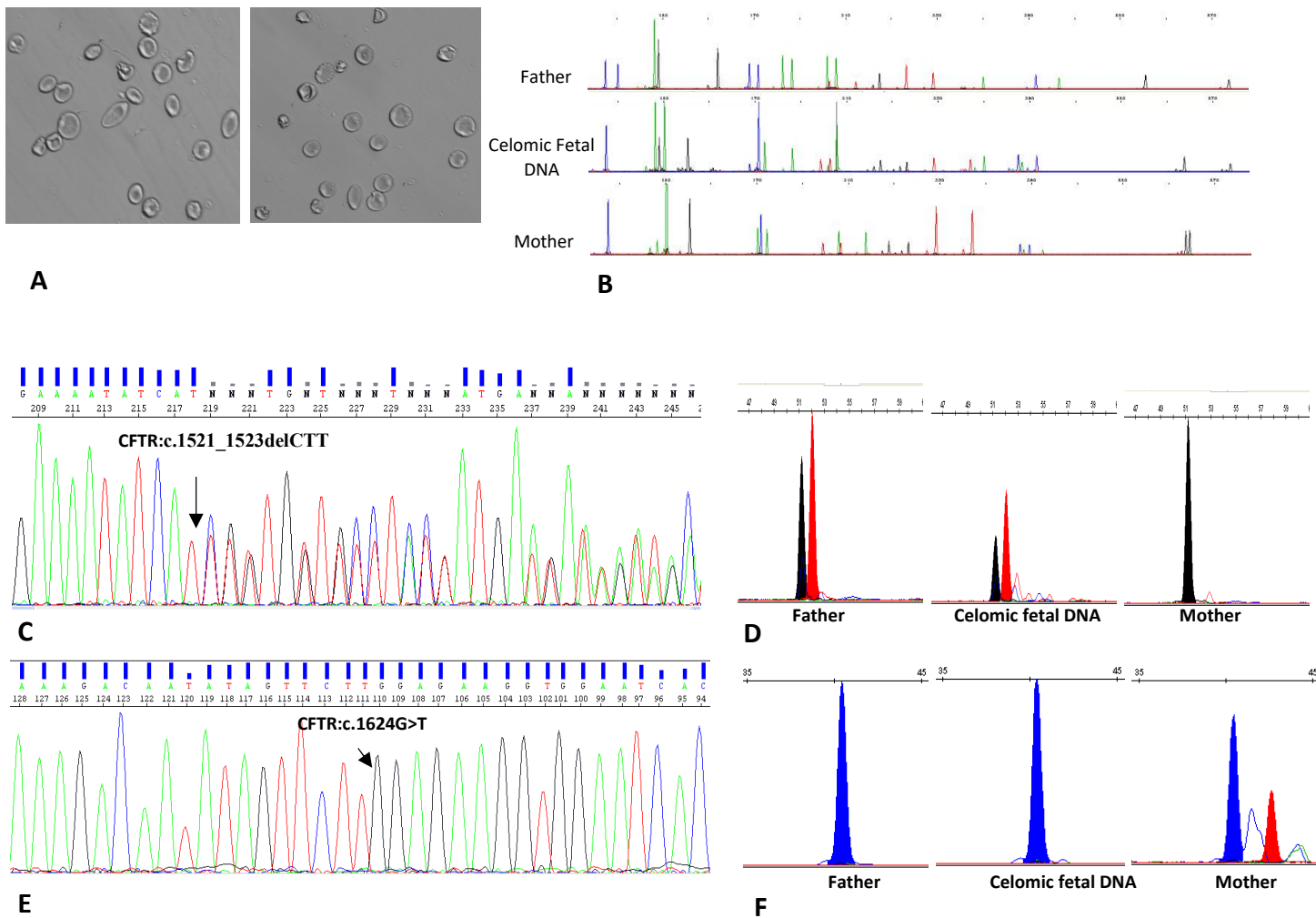

**Figure S5. Case 5.**

Supplement: Supplementary file 1 [file genes-15-00662-s001.zip › Figure S5.pdf]
